# An effective method to reduce lymphatic drainage post-lateral cervical lymph node dissection of differentiated thyroid cancer: a retrospective analysis

**DOI:** 10.1186/s12957-022-02759-z

**Published:** 2022-09-14

**Authors:** Ming-Liang Zhang, Lou-Ming Guo, Peng-Cheng Li, Jing-Kang Zhang, Chen-Xu Guo

**Affiliations:** 1 Department of Oncology Surgery, The First Affiliated Hospital of Bengbu Medical College, No. 287 Changhuai Road, Bengbu, 233003 China; 2 Bengbu Medical College, No. 2600 Donghai Avenue, Bengbu, 233000 China

**Keywords:** Pedicled omohyoid flap covering, Lymph leak, Chyle leak, Lateral cervical lymph node dissection

## Abstract

**Background:**

Lymph or chyle leak (LL/CL) is severe complications after lateral cervical lymph node dissection (LLND), mainly due to iatrogenic injury of the lymphatic duct. Efficient and well-operated methods to reduce postoperative drainage are still lacking. This was a feasibility study to evaluate a new method of preventing LL/CL compared to conventional treatment.

**Method:**

We retrospectively analyzed 20 consecutive patients who used the “pedicled omohyoid flap covering (POFC)” method during LLND from January 2019 to December 2021 in our center as an observation group. Another 20 consecutive patients used the conventional method during LLND in this period as a control group. The clinical and pathological features of the two groups were compared, and the related factors that affected postoperative lymphatic drainage were analyzed with Cox proportional hazards models.

**Results:**

The drainage volume per 24 h and the incidence of LL/CL in the control group were both higher than that in the observation group (all *P* < 0.05), and the number of lymph nodes dissected in the IV region > 10 and the use of the POFC method were the independent risk factors that significantly affected the incidence of LL/CL post LLND (all *P* < 0.05).

**Conclusions:**

POFC is a safe and useful method for reducing drainage and preventing LL/CL post-LLND, especially for patients with heavy metastasis of the lymph nodes in the IV region.

## Introduction

Lateral cervical lymph node dissection (LLND) is a standard procedure for lateral cervical lymph node metastasis (LLNM) diseases, especially in advanced differentiated thyroid carcinoma (DTC). Lymph leak (LL) or chyle leak (CL) are rare but severe complications that could be seen in LLND, mainly caused by iatrogenic injury of lymphatic or thoracic duct [1]. Thoracic or lymphatic duct and their tributary branches are commonly located in the area around the lower internal jugular vein, rendering it prone to inadvertent injury when the lymph nodes need to be thoroughly dissected in this area [2]. A sudden increased daily drainage volume early after LLND is a sign of LL/CL. If it happened, complications associated with it increased significantly, resulting in longer hospital stays, higher treatment costs, and even significant mental and physical trauma (e.g., hypovolemia, malnutrition, electrolyte disturbances, and immunosuppression) [3–5]. Patients with continuous LL/CL who do not relieve after conservative treatment are required to undergo reoperation which may cause further suffering. Moreover, the best conservative treatment and the optimal timing of surgical intervention remain controversial [6–8]. For LL/CL, any postoperative remedial measures are inferior to intraoperative prevention. To overcome these limitations, we try to use an efficient and well-operated method during LLND from January 2020 to reduce postoperative drainage output, which is called “pedicled omohyoid flap covering (POFC).” Good results have been achieved in our clinical practice and are now reported as follows.

## Materials and methods

### Patients

This study was approved by the Ethics Committee of the First Affiliated Hospital of Bengbu Medical College. Forty patients were selected from a consecutive archived cohort of DTC with unilateral LLNM who underwent thyroidectomy and LLND in our center between January 2019 and December 2021. All patients were diagnosed clearly before surgery, with no previous history of neck injury, surgery, and radiotherapy and no other surgical contraindications. They were divided into two groups. Twenty consecutive patients who used POFC method during LLND were regarded as the observation group. The other 20 consecutive patients who used the conventional method during LLND were regarded as the control group. LL was defined as the drainage reaching 200–500 mL/day of clear yellow fluid, and when the volume exceeded 500 mL/day, it was defined as a high output. CL was defined as a fluid in the drainage tube that appears milky white and the triglyceride level is ≥ 0.1 mg/dL by laboratory test regardless of the drainage volume [9]. All patients had a self-satisfaction score at routine follow-up postoperation, 0 for no discomfort and 10 for completely unbearable discomfort. Clinical characteristics and observation variables are shown in Table 1.

**Table 1:** Clinical characteristics and variables of patients

Characteristic	Observation group	Control group	*P* value
Age			
＜55	15	14	0.723
≥55	5	6	
Gender			
male	6	4	0.465
female	14	16	
BMI			
＜24	12	10	0.525
≥24	8	10	
Lymph nodes dissected in IV region			
1~5	6	4	0.726
6~10	12	13	
＞10	2	3	
Histologic			
papillary carcinoma	18	17	0.5
medullary carcinoma	2	3	
Side of dissection			
left	13	17	0.273
right	7	3	
LL/CL			
Positive	0	5	***0.024***
Negative	20	15	
Operation time (mins)	118.5±15.5	113.3±12.4	0.249
Extubation time (days)	6.2±2.3	8.8±2.7	***0.002***
Hospital stay (days)	4.2±1.3	5.8±2.2	***0.008***
Patients’ self-satisfaction score	1.2±0.3	1.8±0.8	***0.017***

### Surgery

**Fig. 1:**
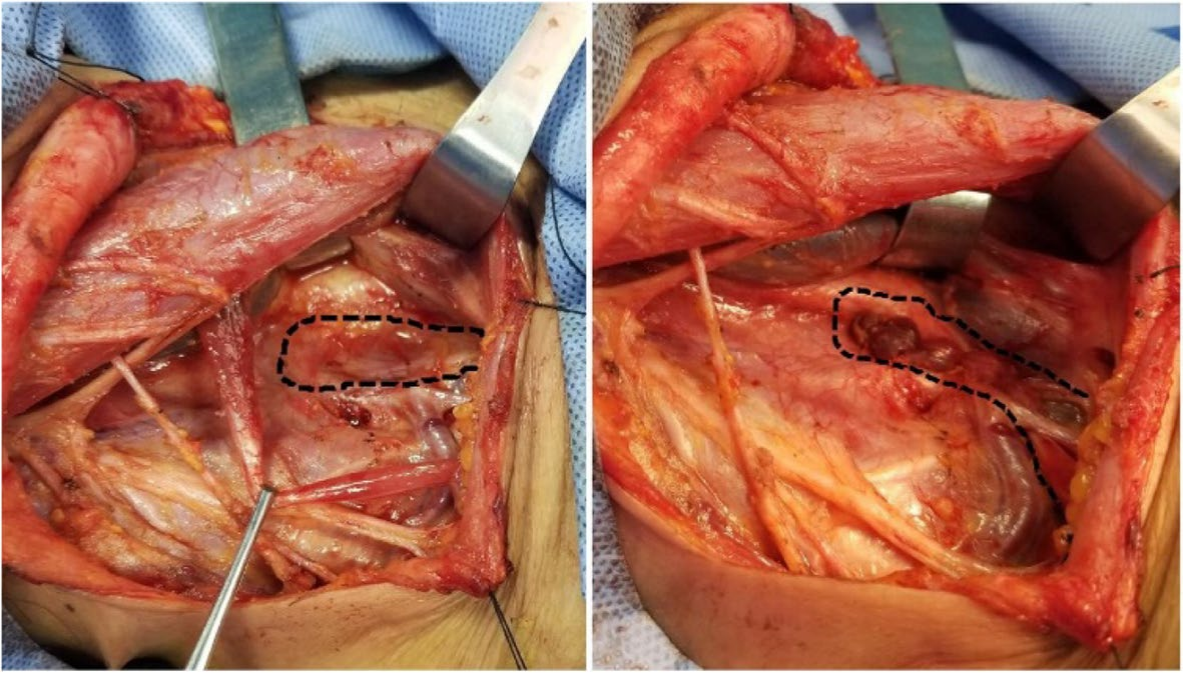
Application of POFC maneuver in lateral cervical lymph node dissection (right)

The LLND procedure was carried out according to the Expert Consensus of Cervical Lymph node Dissection in Differentiated Thyroid Cancer (2017 edition) edited by the Thyroid Surgeons Committee of the Chinese Medical Doctors Association [10]. Surgery was performed under general anesthesia by an experienced oncological thyroid surgeon. The surgery started with the thyroidectomy procedure. Lymph node dissection included levels VI, II, III, IV, and V. Energy surgical instruments (such as high-frequency electrosurgical or ultrasonic knife) were used during surgery. The blood vessel could be occluded by the energy surgical instruments, but the lymphatic vessels could not, because they were composed of a monolayer of endothelial cells and lacked elastic fibers. Careful observation and dissection around the inferior surface of the carotid sheath were necessary to avoid damage thoracic or lymphatic duct. At the end of the lymph node dissection procedures, we asked the anesthesiologist to assist with positive pressure ventilation. If there was no obvious lymph or chylous exudation in the inferior carotid sheath area under observation for at least 1 min, the operation was finished. If there was obvious lymph of chylous exudation during observation, we mainly ligated the injured duct and its tributaries using a running suture (5-0 Prolene) without any other tissue for additional covering [11–13] until the exudation stopped as a conventional method. We found that some patients still had LL/CL after surgery. That might be some small, invisible lymphatic branches having not been occluded [14]. After 2020, we started to use the POFC method: we completely dissociated the omohyoid muscle and severed it from the hyoid attachment, and the scapular tip was retained, then the pedicled muscle flap was folded to cover the level IV region and the branches that might contain residual lymphatic vessels branches around the lower internal jugular vein. The muscle was running sutured with the surrounding soft tissue by 5-0 Prolene for reinforcement (Fig. 1). Negative pressure drainage tube was routinely placed in the vicinity. This method could quickly stop lymphatic or chylous leakage during surgery.

### Statistical analysis

The statistical analyses were performed using a statistical package (SPSS 18.0, Chicago, IL, USA). Paired *T* test was used to compare the *number of lymph nodes dissected in the IV region, operation time, BMI, extubation time, patients’ self-satisfaction score, time of hospital stays, etc.* between the two groups. The chi-square test was used to compare the *incidence of LL/CL* between the two groups. Analysis of variance was used to compare the difference in *drainage volume per day in the first week* between the two groups. Factors that effect on postoperative drainage were analyzed by univariate and multivariable analyses with Cox proportional hazards models. A *P* value < 0.05 was considered statistically significant.

## Results

There were no significant differences in the distribution of age, gender, BMI, number of lymph nodes dissected in level IV region, histological type, side of dissection, and operation times (mean ± SD, 16.5 ± 3.5 min and 15.3 ± 2.4 min) between the observation group and the control group (all *P* > 0.05). The extubation time (mean ± SD, 6.2 ± 2.3 days and 8.8 ± 2.7 days), hospital stay (mean ± SD, 4.2 ± 1.3 days and 5.8 ± 2.2 days), patients’ self-satisfaction scores (mean ± SD, 1.2 ± 0.3 and 1.8 ± 0.8) and incidence of LL/CL between the two groups were statistically different (all *P* < 0.05). The above data of clinical meaning in the observation group were better than that in the control group, which were summarized in Table 1.

**Fig. 2:**
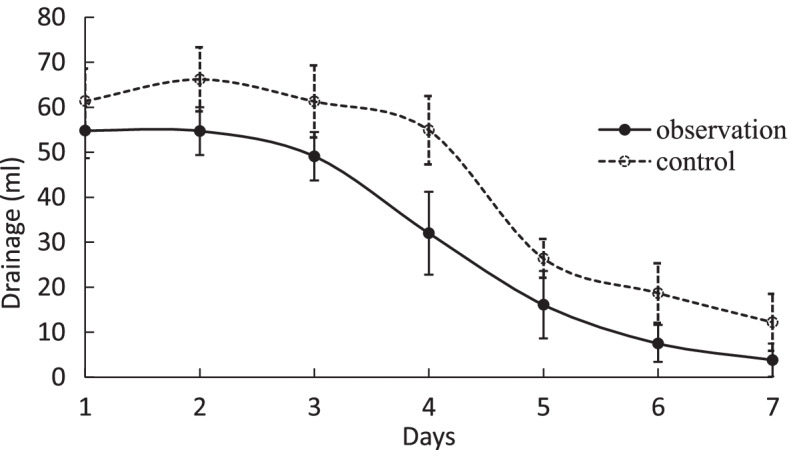
Daily drainage within 1 week after surgery (mL)

Among the 40 patients, the maximum drainage ranged from 15 to 533 mL per 24 h (mean ± SD, 221.37 ± 60.72 mL), and the duration time of leak ranged from 3 to 11 days (mean ± SD, 4.80 ± 2.30 days). The difference in daily drainage between the observation group and the control group was statistically significant (*P* < 0.05), and the maximum drainage volumes were higher in patients in the control group, see Fig. 2.

In addition, 4 (10%) developed LL (1 on the right side, 3 on the left side) and 1 (2.5%) developed CL (on the left side), all happened in the control group; the difference was statistically significant between the two groups (*P* < 0.05, Table 1). The LL began 1–4 days (mean ± SD, 2.75 ± 1.26 days) after surgery, and CL began on the 4th day after surgery. Patients with LL only received negative pressure vacuum (VAC) therapy, while patients with CL received dietary modifications (elimination of long chain triglycerides and use of a medium chain triglyceride diet) based on the VAC treatment. All patients did not use octreotide or somatostatin analogs, and their symptoms were controlled within 10 days. The duration of the leak ranged from 5 to 11 days (mean ± SD, 7.6 ± 2.19 days). Hospital stay post-operation ranged from 5–9 days (mean ± SD, 7.2 ± 1.48 days). Patients’ satisfaction score ranged from 2 to 4 (mean ± SD, 3 ± 0.71) (Table 2).

**Table 2:**
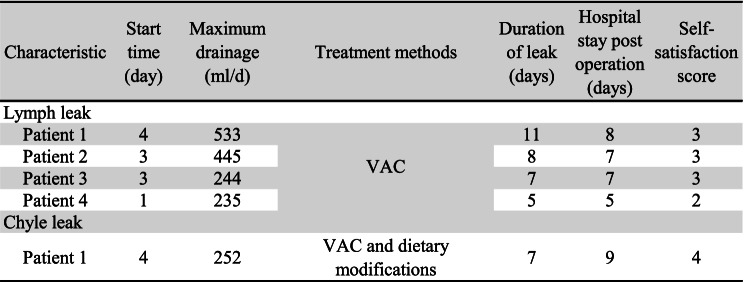
Characteristics of lymph or chyle leakage

Univariate analysis showed that the incidence of LL/CL was not related to gender, age, histologic type, and side of dissection. However, BMI ≥ 24 and lymph nodes dissected in IV region > 10 were the promoting factors for postoperative LL/CL occurrence, while lymph nodes dissected in IV region ≤ 5 and the using of the POFC method could effectively prevent postoperative LL/CL occurrence. Multivariate analysis showed that both the number of lymph nodes dissected in the IV region and using of the POFC method were independent risk factors that significantly affected the incidence of LL/CL post-LLND (Table 3).

**Table 3:**
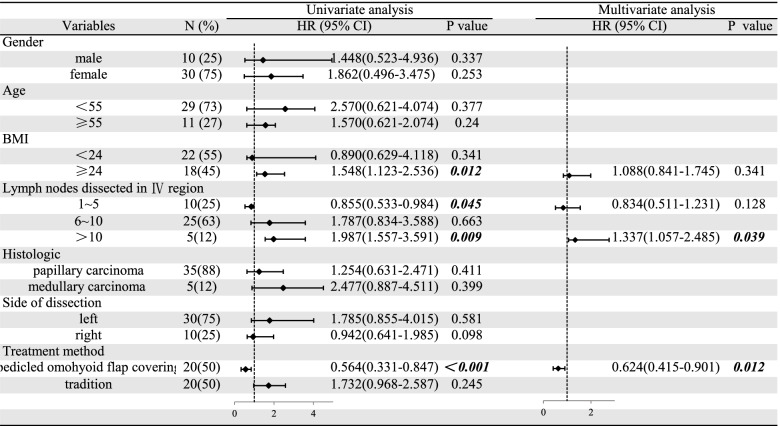
The relationship between clinicopathological factors and incidence of postoperative LL/CL

## Discussion

The thoracic duct drains 3/4 of the systemic lymph, runs in the lower neck that is lateral to the carotid sheath, and terminates on the left venous angle. The remaining 1/4 of the systemic lymph is drained into the right subclavian vein via the lymphatic duct [15–17]. They have different degrees of individual anatomic variation in this region [18]. The anatomic variation and transparent appearance of the lymphatic duct increase the risk of iatrogenic injury during surgery. According to literature statistics, the incidence of increased drainage and lymphatic leak (LL) or chyle leak (CL) caused by cervical lymph node dissection is 5–35% [19–21] and the incidence of serious complications due to CL is about 3–8% [22, 23], which is consistent with the data we reported in this study. Prevention is the key to reduce postoperative complications after LLND. Due to the anatomy of the local IV region, surgeons agree that appropriate management of the level IV region directly affects the incidence of postoperative complications [24].

**Fig. 3:**
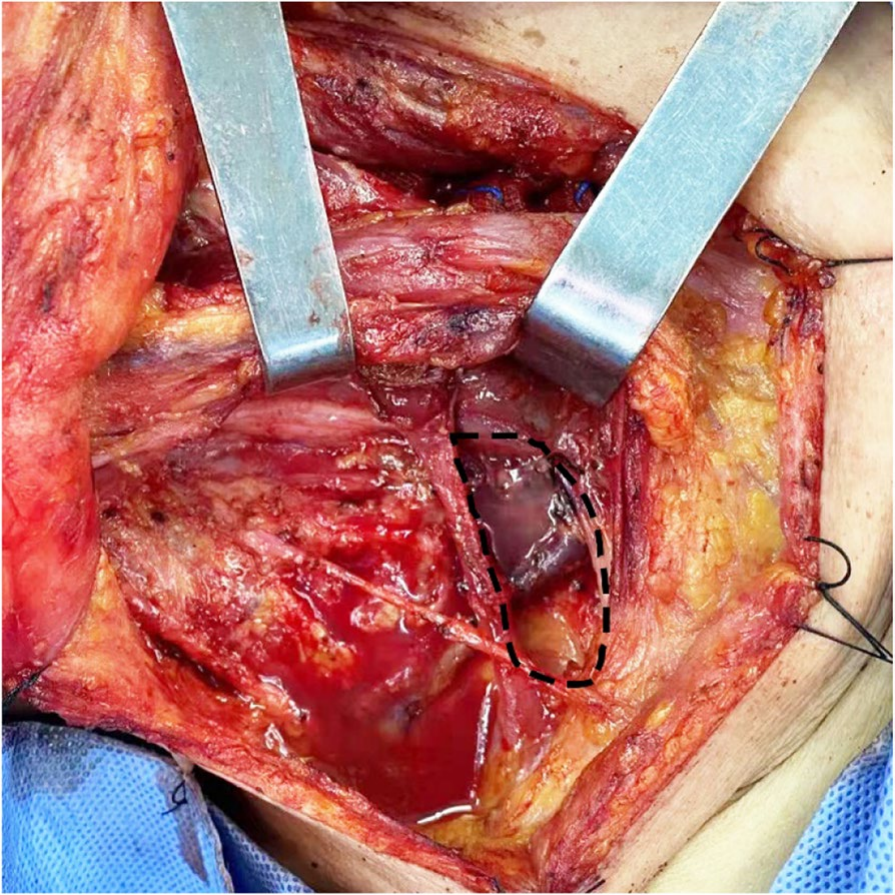
Lymphatic leakage in level IV region during lateral cervical lymph node dissection

The level IV region is one of the most common areas of CLNM in thyroid cancer [25], and meticulous dissection and protection of thoracic or lymphatic ducts during surgery are the main methods to avoid iatrogenic injury. Positive pulmonary pressure ventilation can be performed with the assistance of an anesthesiologist to observe whether there is obvious lymphatic exudation in the IV region after dissection (Fig. 3). Lymphatic vessels are composed of a monolayer of endothelial cells and lack elastic fibers [26]. If no obvious lymphatic drainage is visible, no further treatment is required. Blind suturing may result in tearing of the duct walls, leading to a severe leak. This method, however, may miss the remnants of the smaller lymphatic branches. The small branches are difficult to detect through the eyes under intraoperative anesthesia, and the supraclavicular fossa is not easy to be compressed by gauze after the operation because of its anatomic position. In addition, respiratory, exercise, cough, pain, high-fat diet, and other factors can lead to the lymphatic vessel reopening and the lymphatic drainage fluid increased. LL occurs when the drainage exceeds 200 mL/24 h, usually most obvious on days 2–3 postoperatively [27]. In this study, 4 cases of LL (1 case on the right, 3 cases on the left) and 1 case of CL (1 case on the left) occurred after surgery in the control group. They started 1–4 days after surgery, and all patients were relieved within 11 days after conservative treatment. We found that negative pressure vacuum alone can be used if only LL and the drainage volume < 1000 mL/day; this method had also been reported by other authors [28, 29]. There was no LL/CL happened in the observation group. The routine follow-up patients’ self-satisfaction score in the control group was lower than that in the observation group. The main reason is that extubation time and duration of hospital stay are higher in the control group.

BMI and cervical lymph node metastasis were significantly correlated with the incidence of post-LLND lymphatic leakage [30]. In our study, the univariate analysis found that BMI ≥ 24, the number of lymph nodes dissected in the IV region > 10, and no use of POFC maneuver were risk factors for postoperative LL/CL. Multivariate analysis found that when the number of lymph nodes dissected in IV region > 10, the incidence of LL/CL was significantly increased after the operation. When using the POFC maneuver, the incidence of LL/CL was significantly decreased after the operation. It is suggested that the use of POFC maneuver to cover the bed of level IV region has great clinical significance, especially for patients with heavy-load metastasis of the lymph nodes in this area.

The pedicled omohyoid muscle flap can reach 10–15 cm in length when fully extended. It has enough dimension to cover the area of the inferior lateral carotid sheath and can be sutured with surrounding soft tissues for reinforcement if necessary, blocking off the tiny and imperceptible lymphatic vessels. This extra procedure did not significantly increase the operating time; instead, it gave the surgeon more confidence in preventing complications after LLND. The function of omohyoid muscle in humans is still unclear. Some anatomists believe that its main function is to stabilize the hyoid bone [31]. When the omohyoid muscle is paralyzed or injured on both sides, the swallowing act can cause the hyoid bone to move back-upward, and compression of the carotid artery may cause intracranial pressure changes [32]. However, there are still different views on this statement. In the past, there have been no reports of swallowing discomfort and cerebral blood supply insufficient due to bilateral omohyoid muscle removed in bilateral LLND. Omohyoid muscle is often used as repair and compression material during surgery [33, 34]. In our study, only one side of the omohyoid muscle was severed, and routine follow-up showed no significant discomfort symptoms. The unilateral POFC method can significantly increase patients’ self-satisfaction compared to the complications of LL/CL postoperation. This method is effective, safe, and feasible. Long-term results await longer follow-up data.

## Data Availability

The datasets used or analyzed during the current study are available from the corresponding author upon reasonable request.

## Abbreviations

POFC: Pedicled omohyoid flap covering
LL: Lymph leak
CL: Chyle leak
DTC: Differentiated thyroid cancer
LLNM: Lateral cervical lymph node metastasis
LLND: Lateral cervical lymph node dissection
VAC: Negative pressure vacuum therapy
